# Missed hepatitis b/c or syphilis diagnosis among Kurdish, Russian, and Somali origin migrants in Finland: linking a population-based survey to the national infectious disease register

**DOI:** 10.1186/s12879-018-3041-9

**Published:** 2018-03-20

**Authors:** Paula Tiittala, Matti Ristola, Kirsi Liitsola, Jukka Ollgren, Päivikki Koponen, Heljä-Marja Surcel, Eija Hiltunen-Back, Irja Davidkin, Pia Kivelä

**Affiliations:** 1 Doctoral Programme in Population Health, University of Helsinki, Helsinki, Finland; 2 Department of Health Security, National Institute for Health and Welfare, Mannerheimintie 166, 00271 Helsinki, Finland; 3 Inflammation Center, Helsinki University Hospital, Meilahdentie 2, 00250 Helsinki, Finland; 4 Department of Public Health Solutions, National Institute for Health and Welfare, Mannerheimintie 166, 00271 Helsinki, Finland; 5 Oulu University Hospital, Biobank Borealis of Northern Finland, Aapistie 5B, 90220 Oulu, Finland

**Keywords:** Migrant, Hiv, Hepatitis B, Hepatitis C, Syphilis, Screening, Prevalence, Population-based study, Non-participation, Missed diagnosis

## Abstract

**Background:**

Migrants are considered a key population at risk for sexually transmitted and blood-borne diseases in Europe. Prevalence data to support the design of infectious diseases screening protocols are scarce. We aimed to estimate the prevalence of hepatitis B and C, human immunodefiency virus (HIV) infection and syphilis in specific migrant groups in Finland and to assess risk factors for missed diagnosis.

**Methods:**

A random sample of 3000 Kurdish, Russian, or Somali origin migrants in Finland was invited to a migrant population-based health interview and examination survey during 2010–2012. Participants in the health examination were offered screening for hepatitis B and C, HIV and syphilis. Notification prevalence in the National Infectious Diseases Register (NIDR) was compared between participants and non-participants to assess non-participation. Missed diagnosis was defined as test-positive case in the survey without previous notification in NIDR. Inverse probability weighting was used to correct for non-participation.

**Results:**

Altogether 1000 migrants were screened for infectious diseases. No difference in the notification prevalence among participants and non-participants was observed. Seroprevalence of hepatitis B surface antigen (HBsAg) was 2.3%, hepatitis C antibodies 1.7%, and *Treponema pallidum* antibodies 1.3%. No cases of HIV were identified. Of all test-positive cases, 61% (34/56) had no previous notification in NIDR. 48% of HBsAg, 62.5% of anti-HCV and 84.6% of anti-Trpa positive cases had been missed. Among the Somali population (*n* = 261), prevalence of missed hepatitis B diagnosis was 3.0%. Of the 324 Russian migrants, 3.0% had not been previously diagnosed with hepatitis C and 2.4% had a missed syphilis diagnosis. In multivariable regression model missed diagnosis was associated with migrant origin, living alone, poor self-perceived health, daily smoking, and previous diagnosis of another blood-borne infection.

**Conclusions:**

More than half of chronic hepatitis and syphilis diagnoses had been missed among migrants in Finland. Undiagnosed hepatitis B among Somali migrants implies post-migration transmission that could be prevented by enhanced screening and vaccinations. Rate of missed diagnoses among Russian migrants supports implementation of targeted hepatitis and syphilis screening upon arrival and also in later health care contacts. Coverage and up-take of current screening among migrants should be evaluated.

## Background

Migrants are considered to be especially vulnerable to infectious diseases due to infection epidemiology and conditions in the countries of origin and transit, and behavioural factors and other vulnerabilities [1]. Infectious disease screening upon immigration is one of the most prevalent strategies adopted to address the increased risk for infection [2]. However, recent reports describe considerable post-immigration acquisition of infections, especially human immunodeficiency virus (HIV) [1, 3].

In Finland, 90% of chronic hepatitis B, 14% of hepatitis C, 60% of HIV and 58% of syphilis cases were diagnosed among individuals of foreign origin in 2016 [4]. Since 1990, refugees and asylum seekers to Finland have been offered screening for pulmonary tuberculosis, HIV, hepatitis B, and syphilis on arrival [5]. Screening for hepatitis C is not included in the national screening protocol. In 2010, 4.4% (*n* = 237,066) of the Finnish population had foreign origin and were born abroad [6]. The largest migrant group had Russian origin (23.9%). Somali (*n* = 8368) and Kurdish origin (*n* = 8032) migrants represented the major migrant populations with refugee background in Finland in 2010.

Due to insufficient information on the prevalence of infectious diseases among migrants in Finland, the current screening recommendations are based on the epidemiology of the infectious diseases in the countries of origin [7]. Here, we aimed to assess the prevalence, burden of disease and risk factors for a missed diagnosis of hepatitis B and C, HIV and syphilis in a migrant population-based study validated by a non-participation analysis.

## Methods

The migrant population-based health interview and examination survey (Maamu) examined the health of Kurdish, Russian, and Somali origin migrants in Finland in 2010–2012 [8]. The inclusion criteria for the cross-sectional health interview and examination survey were Kurdish, Russian, or Somali origin, age 18 to 64 years, and having lived in Finland for a minimum of one year. Kurdish origin was defined as speaking Kurdish as the mother tongue and born in Iran or Iraq. Russian origin was defined as speaking Russian or Finnish as the mother tongue and born in Russia or the former Soviet Union (FSU). Individuals born in Somalia were defined as having Somali origin.

A random sample of 3000 migrants, stratified by study location and ethnicity, was drawn from the Population Information System (PIS). All residents in Finland are registered to the Finnish PIS and given a unique Personal Identity Code (PIC). Temporary residents, such as travelers or asylum seekers, are registered a temporarory PIC while accessing public services. After receiving residency, a temporary PIC is replaced by a permanent PIC in electronic health records or national registers.

**Fig. 1:**
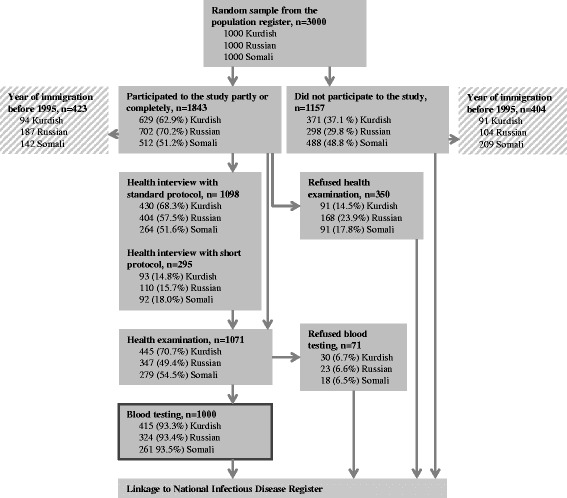
Study participation

One thousand migrants from each ethnic group were invited to participate in a health interview followed by a health examination (Fig. 1). Those who refused the 281-item standard protocol were offered the possibility to participate in a short interview containing only the most relevant 62 questions, or in health examination only. During the health interview the participants were questioned about previous history of infectious diseases and participation in screening for infectious diseases upon arrival to Finland, among other socio-demographic determinants, including mental health symptoms [9]. Participants provided a written informed consent separately for participation in the health interview, examination, and testing.

Screening tests were offered during the health examination. Hepatitis B surface antigen (HBsAg) was tested with Abbot ARCHITECT® HBsAg (Abbot, Chigago, Illinois,USA) and confirmed with Abbot AXSYM® Confirmatory. Hepatitis C antibodies (anti-HCV) were tested with Abbot ARCHITECT® Anti-HCV and confirmed with Fujirebio INNOTEST® HCV Ab (Fujirebio, Malvern, Pennsylvania, USA). Serum HIV antigen and antibodies (HIVAgAb) were tested with Abbot ARCHITECT® HIV Ag/Ab Combo and confirmed with Fujirebio INNO-LIA® HIV. Treponema pallidum specific antibodies (anti-Trpa) were tested with Abbot ARCHITECT® TP and confirmed with Fujirebio INNO-LIA® Syphilis. The laboratory analyses were performed in the National Institute for Health and Welfare (THL) [10]. Anti-HCV seropositivity was confirmed in HUSLAB (Helsinki, Finland). All those who participated in testing were informed about the results. Negative results were sent in mailed letters and positive findings were communicated by a study physician who also referred the patients for follow-up.

The National Infectious Diseases Register (NIDR) was established on 1 January 1995. Both physicians and laboratories are obliged by the Communicable Disease Act to report specific diagnoses and microbiological findings to the NIDR. Their reporting is independent of each other. We linked the Maamu sample to the NIDR using the PICs and retrieved notifications for HIV, hepatitis B and C, and syphilis that were made before the launch of the Maamu study on 1 January 2010. For the analysis, we excluded those migrants who had immigrated to Finland before January 1995 and who could have had a diagnosis without notification to the NIDR. To evaluate the impact of non-participation, we compared the notification prevalence in the NIDR between participants and non-participants during 1 January 1995 and 31 December 2009. Register linkage did not require a consent from the non-participants. A case testing positive in the Maamu study, but without previous notification to the NIDR was considered a “missed” diagnosis.

### Statistical methods

IBM SPSS Statistics 24 (Microsoft), including a Complex Samples module, was used for the statistical analysis. The multiple choice variables were recoded into two categories. Non-participation was addressed by calculating inverse probability weights for all participants [8, 11]. Factors associating with participation were assessed with logistic regression and, based on Bayeasian Information Criterion, age group, sex, migrant group, municipality, and marital status were chosen as auxiliary variables in the weight calculation. Separate weights were developed for participation in different stages of the study: the health interview with the standard or short protocol, or in the health examination. Non-participants were assigned a zero weight.. Null hypothesis was tested using Wald’s F-test and *p*-values below 0.05 were considered significant. For the multivariable logistic regression, variables with a univariate p below 0.2 were included. The interactions between variables in the multivariate model were tested. All individuals who participated in testing were included in the multivariable model. The odds ratios (ORs) for continuous variables were calculated for every one year increase.

## Results

### Basic characteristics

Altogether 1843 individuals, representing 61.4% of the sample, participated in the Maamu study (Fig. 1). Of these, 423 participants had immigrated before 1 January 1995 and were excluded from the analysis. The overall participation in the study was highest among Russian migrants, but a large proportion of these individuals had moved to Finland before 1995. The study population comprised 1000 participants who were tested for an infectious disease. The non-participants to the study and the individuals who refused the health examination or the blood testing were considered non-participants in the analysis.

The overall acceptability of testing did not differ between Kurdish, Russian, or Somali participants (93.3%, 93.4%, and 93.5%, respectively, *p* = 0.988). HIVAgAb test was accepted by 91.7% of the participants to the health examination without a difference between the migrant groups (*p* = 0.433). There was also no difference in the acceptability of HBsAg and anti-HCV tests (93.2%, *p* = 0.997) or the anti-Trpa test (92.3%, *p* = 0.825) among participants of different origin.

Migrants from Somalia were significantly younger (mean 32.4 years) than Kurdish (34.9 years) or Russian (38.6 years) migrants (Table 1). In general, men were younger (mean 34.5 years) than women (mean 36.4 years) (*p* = 0.027) and men had lived in the country for a shorter period (mean 8.7 years) than women (mean 9.4 years) (*p* < 0.001). Russian migrants were older at immigration (mean 29.6 years) than Kurdish (25.5 years) or Somali (23.6 years) migrants. Twice as many Somali immigrants had arrived to Finland before the age of 18 years relative to Kurdish or Russian migrants. Two thirds (67.2%) lived in the capital area. Especially Kurdish and Somali men had received their residency permit as refugees or due to international protection of asylum seekers.

**Table 1:** Population weighted^a^ basic characteristics of the the study population; % (crude n)

Characteristic	Total; n = 1000				Men; *n* = 447				Women; *n* = 553			
	Kurdish; *n* = 415	Russian; *n* = 324	Somali; *n* = 216	p	Kurdish; *n* = 225	Russian; *n* = 119	Somali; *n* = 103	p	Kurdish; *n* = 190	Russian; *n* = 205	Somali; n = 261	p
Age												
18–29 years 30–44 years 45–64 years	34.1 (141) 47.2 (193) 18.7 (81)	29.1 (92) 34.1 (111) 36.8 (121)	46.0 (123) 37.0 (85) 16.9 (53)	**< 0.001**	36.1 (81) 46.2 (102) 17.7 (42)	31.7 (35) 39.5 (47) 28.8 (37)	53.5 (59) 31.4 (28) 15.1 (16)	**0.009**	31.6 (60) 48.4 (91) 20.0 (39)	27.5 (57) 30.7 (64) 41.8 (84)	41.4 (64) 40.5 (57) 18.1 (37)	**< 0.001**
Age at immigration												
Below 18 years 18 to 30 years 31 years or more	18.5 (84) 51.0 (204) 30.5 (127)	19.2 (60) 35.0 (111) 45.8 (153)	36.4 (106) 39.5 (87) 24.1 (68)	**< 0.001**	16.9 (47) 53.5 (111) 29.7 (67)	28.3 (32) 30.0 (34) 41.7 (53)	45.3 (52) 32.0 (29) 22.7 (22)	**< 0.001**	20.6 (37) 47.9 (93) 31.5 (60)	13.6 (28) 38.1 (77) 48.3 (100)	30.9 (54) 44.2 (58) 24.9 (46)	**< 0.001**
Time of residency in Finland												
Less than 5 years 5–10 years 11 years or more	23.2 (97) 36.9 (145) 39.8 (160)	29.4 (98) 29.5 (93) 41.1 (128)	31.6 (95) 32.7 (75) 35.6 (76)	0.079	24.5 (55) 40.7 (84) 34.9 (74)	27.1 (35) 35.1 (39) 37.8 (42)	34.4 (41) 41.6 (33) 24.0 (22)	0.264	21.7 (42) 32.3 (61) 46.0 (86)	30.8 (63) 26.1 (54) 43.1 (86)	29.9 (54) 27.2 (42) 42.9 (54)	0.280
Living in the Helsinki metropolitan area	47.4 (189)	79.9 (204)	83.8 (153)	**< 0.001**	45.1 (91)	79.4 (72)	78.5 (51)	**< 0.001**	50.3 (98)	80.3 (132)	87.0 (102)	**< 0.001**
Immigration as an asylum seeker or refugee	73.2 (277)	1.2 (3)	72.0 (151)	**< 0.001**	85.2 (171)	1.7 (2)	93.5 (75)	**< 0.001**	58.8 (106)	0.9 (1)	56.7 (76)	**< 0.001**
Married, registered relationship or co-habitation	66.4 (278)	58.5 (200)	66.2 (161)	0.089	63.5 (140)	56.5 (71)	58.3 (55)	0.465	70.0 (138)	59.7 (129)	71.1 (106)	0.060
Secondary education or more^b^	41.7 (162)	78.5 (249)	18.8 (36)	**< 0.001**	42.3 (86)	72.6 (82)	30.7 (22)	**< 0.001**	41.0 (76)	82.2 (167)	11.3 (14)	**< 0.001**
Employed, full or part-time	37.7 (147)	47.8 (152)	16.1 (36)	**< 0.001**	44.7 (93)	53.3 (61)	20.7 (19)	**< 0.001**	28.9 (54)	44.3 (91)	13.2 (17)	**< 0.001**
Able to read well in Finnish/ Swedish^c^	83.5 (308)	83.7 (252)	85.0 (154)	0.893	85.3 (167)	83.9 (89)	93.4 (77)	0.112	81.2 (141)	83.5 (163)	77.9 (77)	0.529
Experienced violence during the last 12 month	6.5 (Z7)	1.2 (4)	3.9 (8)	**0.006**	6.5 (12)	1.2 (1)	0 (0)	**0.007**	6.4 (15)	1.1 (3)	6.7 (8)	**0.014**

Bolded p-values represent statistically significant differences between the migrant groups

^a^Population weights developed according to age group, sex, migrant population, municipality and marital status

^b^Highschool, professionnal school or similar

^c^Two categories of a 4 gategory Likert scale combined: moderate or good reading skills

Russian immigrants had higher education than migrants of Kurdish or Somali origin and were more often employed. However, no difference in self-perceived literacy in Finnish or Swedish was observed. Somali women had significantly less tertiary education than men (11.3% vs. 30.7%, *p* = 0.002) and had poorer literacy skills (77.9% vs. 93.4%, *p* = 0.004).

Somali migrants perceived their health as significantly better than Kurdish or Russian participants and reported less long-term illnesses. Migrant women in general reported less often a good health status than men (66.6% vs. 78.3%, *p* < 0.001) and reported more chronic illnesses (33.9% vs. 23.0%, p < 0.001). Psychiatric symptoms were reported especially by Kurdish women.

### Health behaviours

Important differences emerged in health behaviours and health care seeking practices between the migrant groups (Table 2). Somali migrants did not report any alcohol consumption during the past year. Among Kurdish migrants, a significantly smaller proportion of women than men had used alcohol within the last year (p < 0.001). Smoking was significantly more common among migrant men (25.0%) than women (4.1%, p < 0.001). Only two participants reported previous use of injectable drugs.

**Table 2:** Population weighted^a^ health status and health behaviours of the study population; % (crude n)

Characteristics	Total; n = 1000				Men; n = 447				Women; n = 553			
	Kurdish; n = 415	Russian; n = 324	Somali; n = 216	p	Kurdish; n = 225	Russian; n = 119	Somali; n = 103	p	Kurdish; n = 190	Russian; n = 205	Somali; n = 261	p
Long term illness	31.5 (123)	35.3 (114)	16.1 (35)	**< 0.001**	28.9 (58)	23.1 (29)	8.3 (8)	**0.003**	34.6 (65)	42.8 (85)	21.0 (27)	**0.001**
Self-perceived good health	68.1 (271)	66.4 (206)	85.9 (212)	**< 0.001**	72.7 (156)	77.4 (85)	93.2 (89)	**0.003**	62.5 (115)	59.5 (121)	81.3 (123)	**< 0.001**
Mental health symptoms^b^	34.4 (138)	15.7 (50)	10.2 (28)	**< 0.001**	22.6 (45)	8.8 (10)	7.0 (8)	**0.001**	48.8 (93)	19.9 (40)	12.1 (20)	**< 0.001**
At least one previous delivery (women)	NA	NA	NA	NA	NA	NA	NA	NA	78.8 (145)	71.5 (142)	78.7 (96)	0.247
At least one previous abortion (women)	NA	NA	NA	NA	NA	NA	NA	NA	22.4 (39)	55.8 (106)	1.3 (4)	**< 0.001**
Alcohol consumption during the last 12 months	37.0 (143)	87.1 (277)	0 (0)	NA	53.2 (110)	87.6 (100)	0 (0)	NA	17.1 (33)	86.8 (177)	0 (0)	NA
High alcohol consuption (AUDIT-C)^c^	1.0 (6)	3.2 (10)	0 (0)	NA^d^	1.9 (6)	5.2 (8)	0 (0)	NA	0 (0)	2.0 (2)	0 (0)	NA^d^
Smokes daily	18.2 (71)	16.3 (50)	2.1 (9)	**< 0.001**	30.5 (64)	29.6 (33)	5.4 (9)	**< 0.001**	3.2 (7)	8.1 (17)	0 (0)	NA^d^
History of injecting drugs	0.2 (1)	0.3 (1)	0	NA	0.4 (1)	0.7 (1)	0 (0)	0.628	0 (0)	0 (0)	0 (0)	NA
Visited a doctor during the last 12 months	70.3 (283)	69.1 (224)	56.3 (131)	**0.002**	67.4 (143)	59.1 (73)	52.7 (46)	0.059	73.8 (140)	75.4 (151)	58.6 (85)	**0.005**
Health check-up during the past 5 years	72.6 (266)	79.2 (246)	59.9 (114)	**< 0.001**	68.8 (131)	67.1 (79)	63.7 (50)	0.734	77.2 (135)	87.1 (167)	56.9 (64)	**< 0.001**
Healthcare visit due to a mental health problem during the last 12 months	11.7 (49)	4.1 (16)	1.7 (4)	**< 0.001**	5.5 (10)	5.8 (7)	0.6 (1)	0.056	19.2 (39)	3.0 (9)	2.4 (3)	**< 0.001**
Participation to infectious diseases screening upon arrival to Finland	84.9 (324)	13.8 (61)	80.9 (172)	**< 0.001**	85.7 (173)	17.8 (30)	84.4 (72)	**< 0.001**	84.0 (151)	11.3 (31)	78.4 (100)	**< 0.001**
Post-migration travelling to country of origin	59.6 (229)	97.5 (300)	15.7 (36)	**< 0.001**	53.4 (106)	98.6 (110)	9.1 (10)	**< 0.001**	67.2 (123)	96.8 (190)	20.6 (26)	**< 0.001**
Previos diagnosis of a blood borne infection	0.6 (2)	4.6 (15)	14.4 (36)	**< 0.001**	0.7 (1)	5.3 (6)	12.8 (14)	**0.004**	0.6 (1)	4.1 (9)	15.5 (22)	**< 0.001**

Bolded *p*-values represent statistically significant differences between the migrant groups

*NA* Not available

^a^Population weights developed according to age group, sex, migrant population, municipality and marital status

^b^Hopkins Sympton Checklist-25 (HSCL-25) score over 1.75

^c^AUDIT-C is a validated, three question tool to screen unhealthy alcohol use. Positive score for 7 points or more

^d^A quasi-complete segregation in the data. The maximum likelihood estimation does not exist

Migrants from Somalia had visited a doctor during the last year or participated in a health check-up during the last five years significantly less often than Kurdish or Russian migrants (*p* = 0.002 and < 0.001, respectively). More Russian women reported visiting a doctor within the past year (75.4% vs. 59.1%) and participating in health check-ups during the last five years (87.1% vs. 67.1%) than Russian men (*p* = 0.007 and < 0.001, respectively). Somali women had significantly less abortions than Kurdish or Russian migrants.

Post-migration travelling to the former country of origin was especially prevalent among Kurdish and Russian migrants. Men reported more travelling than women (68.0% vs. 57.8%, *p* = 0.003). As a reflection of the migration background, Kurdish and Somali origin migrants reported more often participation in infectious disease screening upon arrival to Finland. Somali migrants reported more often a previous diagnosis of a blood-borne infection.

### Non-participation

Notification prevalence in the NIDR between 1 January 1995 and 31 December 2009 did not differ between participants and non-participants with respect to hepatitis B or C or syphilis (*p* = 0.387, 0.992, and 0.586, respectively). No previous notifications of HIV were identified among the participants or among the non-participants to the testing.

### Seroprevalence and missed diagnosis

The overall HBsAg seroprevalence was 2.3% [95% CI 1.5–3.5%], the seroprevalence of anti-HCV was 1.7% [1.0–2.9%], and the seropositivity of anti-Trpa was 1.3% [0.7–2.3%]. No HIVAgAb-positive cases were identified. Significant differences in disease prevalence were observed between and within migrant groups (Table 3). Seropositivity to HBsAg was highest among the Somali migrant population, whereas anti-HCV and Trpa-Ab were most prevalent among the Russian migrant population. Among subjects of Russian origin, women had a significantly higher rate of anti-Trpa positivity than men (*p* = 0.044). None of the Kurdish women tested positive for HBsAg, anti-HCV or anti-Trpa.

**Table 3:** Population weighted^a^ prevalence of infectious diseases among the study population; % [95% Confidence Interval] (crude n). Total n = 1000

	Kurdish; n = 415		Russian; n = 324		Somali; n = 261		p
	n	% (95% CI)	n	% (95% CI)	n	% (95% CI)	
HIVAgAb; *n* = 982	0	0	0	0	0	0	NA
HBsAg; *n* = 996	2	0.4 (0.1–1.5)	5	1.7 (0.7–4.3)	16	6.0 (3.6–9.9)	**< 0.001**
anti-HCV; n = 996	1	0.1 (0.0–0.5)	12	4.1 (2.2–7.3)	3	1.3 (0.4–4.1)	**< 0.001**
anti-Trpa; *n* = 988	1	0.3 (0.1–1.9)	9	2.9 (1.5–5.6)	2	1.0 (0.2–3.8)	**0.042**

Bolded p-values represent statistically significant differences between the migrant populations

*NA* Not available

^a^Population weights developed according to age group, sex, migrant population, municipality and marital status

The infectious disease prevalence increased consistently with age. The youngest age cohort (18–29 years) had an HBsAg prevalence of 1.8% [95% CI 0.8–4.0%], the 30- to 44-year-olds 2.1% [1.0–4.3%], and the 45- to 64-year-olds 3.4% [1.7–6.5%]. A similar observation was made for anti-HCV seroprevalence (0.7% [0.2–3.0%] among the youngest, 1.5% [0.6–3.8%] among the middle group, and 3.5% [1.8–6.8%] among the oldest group) and for anti-Trpa seroprevalence (0.4% [0.1–2.1%] among the youngest, 1.2% [0.4–3.2%] among the middle group, and 3.1% [1.5–6.4%] among the oldest group).

**Fig. 2:**
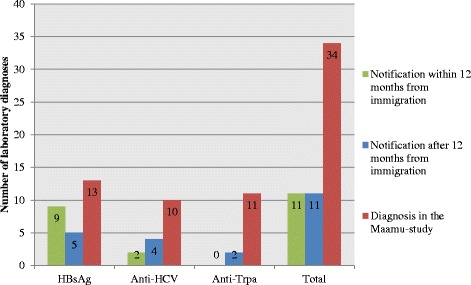
Delay from immigration to diagnosis among the study population; *n* = 1000

Altogether 60.7% (*n* = 34) of the laboratory diagnoses made in the Maamu study had no previous notification in the NIDR and were considered missed diagnoses (Fig. 2). These 34 missed laboratory diagnoses were identified in 33 individuals. The majority of the anti-HCV (62.5%, *n* = 10) and anti-Trpa (84.6%, *n* = 11) findings had no previous notification in the NIDR, whereas for HBsAg 48.1% (*n* = 13) had not been diagnosed earlier. Of the previously made diagnoses, 50.0% were notified within a year of the immigration.

The highest number of missed diagnoses was observed among Russian migrants, 5.9% (*n* = 18) of whom were diagnosed with a previously unnotified infectious disease. Of these, 3 cases tested positive for HBsAg, 7 cases for anti-HCV, and 7 cases for anti-Trpa. One case tested positive for both anti-HCV and anti-Trpa. Of the Somali migrants, 4.1% (n = 11) tested positive for a previously unnotified infectious disease. Of these, 8 cases tested positive for HBsAg, 1 case for anti-HCV, and 2 cases for anti-Trpa. The Kurdish migrants had the lowest number of missed diagnoses (0.9%, *n* = 4). Of these, 2 were HBsAg, 1 anti-HCV, and 1 anti-Trpa. Eight cases with a missed diagnosis reported that they had been previously diagnosed with a blood-borne infection and six of them named the disease correctly.

Missed diagnosis was associated with living alone, self-perceived poor health, smoking cigarettes, and self-reported previous diagnosis of a blood-borne infection (Table 4). A significant interaction was observed between origin and marital status, as married Somali origin migrants had a significantly lower risk for a missed diagnosis than Somali origin migrants who lived alone. Additionally, in univariate analysis, missed diagnosis was associated with Somali or Russian origin, older age at time of study and time of immigration, no self-reported long-term illness, history of injecting drug use (OR 27.728, 95% CI 1.94–395.50), consumption of alcohol during the last 12 months (OR 2.98, 95% CI 1.29–6.90), and for women, having had at least one previous abortion (OR 3.23, 95% CI 1.11–9.53). Due to quasi-complete separation in the data, history of previous injecting drug use, alcohol consumption within the last 12 months and at least one previous abortion were excluded from the multivariable analysis.

**Table 4:** Risk factors for a missed HBsAg, anti-HCV or anti-Trpa diagnosis

Variable		Missed diagnosis; %^a^ (n^b^)	Univariable p	Missed diagnosis; OR (95% CI)	Multivariable p	Missed diagnosis; aOR (95% CI)
Origin						
	Kurdish Russian Somali	0.9 (4) 5.9 (18) 4.1 (11)	**0.001**	**1** **7.36 (2.51–21.60)** **5.26 (1.66–16.65)**	NA	1 **7.74 (1.55–38.75)** 1.52 (0.18–13.10)
Age^c^						
	18-29 years 30–44 years 45–64 years	1.2 (6) 2.9 (9) 7.2 (18)	**< 0.001**	**1.06 (1.03–1.09)**	0.816	0.989 (0.90–1.07)
Age at immigration^c^						
	Below 18 years 18 to 30 years 31 years or more	2.1 (8) 1.2 (4) 6.9 (21)	**< 0.001**	**1.05 (1.02–1.09)**	0.214	1.061 (0.97–1.16)
Married, registered relationship or co-habitation						
	Kurdish Russian Somali	0.6 (2) 8.6 (11) 6.8 (3)	0.393 0.734 **0.012**	0.43 (0.06–3.01) 1.19 (0.43–3.32) **0.15 (0.03–0.65)**	**0.001**	0.37 (0.05–2.65) 1.05 (0.34–3.22) **0.05 (0.01–0.36)**
Long term illness		5.4 (13)	**0.044**	**0.46 (0.22–0.98)**	0.861	0.91 (0.32–2.58)
Perceived good health		2.1 (15)	**0.003**	**0.32 (0.15–0.67)**	**0.064**	**0.34 (0.11–1.06)**
Smokes daily		6.8 (9)	**0.025**	**2.57 (1.13–5.87)**	**0.003**	**3.71 (1.59–8.68)**
Previos diagnosis of a blood borne infection		13.9 (8)	**< 0.001**	**5.78 (2.30–14.50)**	**0.004**	**9.00 (2.05–39.44)**

Bolded *p*-values represent statistically significant differences in prevalence of missed diagnosis

^a^Weighted prevalence, Population weights developed according to age group, sex, migrant population, municipality and marital status

^b^Crude n

^c^Variable used as a continuous variable in the univariate and multivariate analysis

### Burden of infections

Based on the number of first-generation migrants in Finland in 2016 [6], we extrapolated the number of missed hepatitis B or C or syphilis diagnoses among the 20- to 64-year-old migrant populations (Table 5). The largest burden of missed infections was estimated among the Russian migrant population, with altogether 3538 missed hepatitis B or C or syphilis diagnoses.

**Table 5:** Estimated burden of missed hepatitis B/C and syphilis diagnoses among 20–64 year old migrant populations in Finland

		Kurdish^a^	Russian^b^	Somali
Migrant population in Finland^a^; aged 20 to 64 years; n		17,000	58,000	9000
Migrant population tested in Maamu; n		415	324	261
Hepatitis B				
	Prevalence of missed HBsAg diagnosis in Maamu; %^c^ [95% CI] (n^d^)	0.4 [0.1–1.5] (2)	0.7 [0.2–2.2] (3)	3.0 [1.4–6.1] (8)
	Number of missed HBsAg diagnosis in the population; n [95% CI]	68 [17–255]	406 [116–1276]	270 [126–549]
Hepatitis C				
	Prevalence of missed anti-HCV diagnosis in Maamu; %^c^ [95% CI] (n^d^)	0.1 [0.0–0.5] (1)	3.0 [1.5–5.9] (8)	0.3 [0.0–1.9] (1)
	Number of missed anti-HCV diagnosis in the population; n [95% CI]	17 [0–85]	1740 [870–3422]	27 [0–171]
Syphilis				
	Prevalence of missed anti-Trpa diagnosis in Maamu; %^c^ [95% CI] (n^d^)	0.3 [0.1–1.9] (1)	2.4 [1.2–4.9] (8)	1.0 [0.2–3.8] (2)
	Number of missed anti-Trpa diagnosis in the population; n [95% CI]	51 [17–323]	1392 [696–2842]	90 [18–342]

^a^First generation immigrants with a country of origin Iraq or Islamic Republic of Iran

^b^First generation immigrants with a country of origin Russia or Former Soviet Union

^c^Weighted prevalence. Population weights developed according to age group, sex, migrant population, municipality and marital status

^d^Crude n

## Discussion

To our knowledge, the Maamu study was the first migrant population-based study to assess the prevalence of HIV, hepatitis B and C, and syphilis and associated risk factors among unselected migrant populations. The majority of the infections identified in the study had not been previously notified and were hence considered “missed”. Findings from the non-participation analysis imply that the results from the study are generalizable to the total migrant populations. Population-based observations of infectious disease prevalence among unselected migrant populations are scarce [12, 13], as many studies have recruited migrants with presumably higher baseline risk for infection, e.g. from health care settings or with limited access to services [14].

The infection profile differed significantly between Kurdish, Russian, and Somali migrant populations. The highest HBsAg prevalence was identified among Somali migrants, whereas the highest prevalences of hepatitis C and syphilis were observed among Russian migrants. Kurdish migrants had low levels of infections. The identified hepatitis B/C seroprevalence rates were in general comparable [13, 15] or lower [16] than those reported in previous literature among migrants to high-income countries [17]. Similarly was the prevalence lower or comparable than among the general population in the countries of origin. Hepatitis B seroprevalence in Iran has been estimated at 2.8–3.7%, in Russia and FSU 2.3–5.6% and in Somalia 9.1–19.8% [18]. A modelling study estimated the prevalence of chronic viremic hepatitis C in Iran and Iraq at 0.2–0.3% and in Russia at 2.3–3.5% [19]. Hepatitis C antibody prevalence in Somalia has been estimated at 0.3–1.9% [20].

Syphilis is considered not to disproportionately infect migrants in the European region [1]. Only a minority of the European countries, including Finland, have included syphilis in the national screening recommendations [2, 5]. Our results demonstrate noteable syphilis prevalence among unselected Russian and Somali migrant populations, with the majority of syphilis infections remaining undiagnosed. The findings suggest that migrants have vulnerabilities to syphilis and support the inclusion of syphilis in the national screening protocols.

No cases of HIV were observed in our study, which can be explained by selection of migrants originating from low prevalence settings [21]; the HIV prevalence among adults in Somalia has been estimated at 0.2–0.5% and in Iran at < 0.1–0.2% in 2016 [22]. Estimates of the HIV prevalence in the general population in Russia are scarce as studies tend to assess key populations at risk for HIV [23]. HIV prevalence among adults in the region has been estimated at 0.8–1.0% [24]. Concentration of the epidemic to people who inject drugs and prisoners might explain the observed low prevalence of HIV among Russian origin migrants in Finland.

Seroprevalence of HBsAg, anti-HCV, and anti-Trpa increased consistently with age in all migrant populations, in line with earlier studies [16]. Missed diagnosis was associated with older age at testing and older age at immigration in the univariate analysis. Older age at migration results in a prolonged exposure time in high prevalence settings. In our study, Russian origin migrants had immigrated to Finland at a significantly older age, and hence, their exposure time in higher prevalence settings had been longer.

The proportion of missed diagnoses was considerable. Missed hepatitis B infection was especially prevalent among Somali migrants, where half (8/16) of the HBsAg findings had not been diagnosed previously. Among Russian migrants, the majority of hepatitis B (3/5), hepatitis C (7/12), and syphilis (8/9) diagnoses had been missed. Reasons for missed diagnosis include no screening on arrival and post-migration infections. A late diagnosis increases the morbidity and complications related to a disease and may contribute to the spread of infections. Considering that 6–25% of the migrants from hepatitis C endemic countries are estimated to be viremic [17], the risk of further transmitting the infection is important.

Missed diagnosis was associated with factors that could explain health behaviours or service use that increases the risk for infection or missed diagnosis, i.e. not living with a partner, perceived poor health, and smoking. Similarly, as observed in the univariate analysis, previous abortion and alcohol use might be related to risky sexual behaviour or addictions. Additionally, missed diagnosis was associated with self-reported previous diagnosis of a blood-borne infectious disease. Individuals who reported a previous diagnosis, but for whom the diagnosis had not been confirmed in Finland are beyond the reach of the health care system, treatment, and preventive measures. Surprisingly, previous delivery was not associated with a lower risk for missed diagnosis, even though all pregnant women in Finland have been offered screening for HIV (since 1998), hepatitis B (since 1994), and syphilis (since the 1950s) and the up-take of antenatal screening is 98% [10]. However, the up-take and coverage of antenatal screening has not been evaluated among migrant populations [25]. Furthermore, we were not able to distinguish births in Finland from those in other countries.

Three out of four Somali migrants had arrived to Finland as a refugee or an asylum seeker, and hence, should have been offered a health check-up and infectious disease screening upon arrival. Self-reported participation in infectious disease screening upon immigration did not influence the risk for missed diagnosis. More Kurdish and Somali migrants reported having participated in infectious disease screening upon arrival (85% and 81%, respectively) than had arrived to the country as a refugee or an asylum seeker (73% and 72%, respectively). This over-reporting is explained by a portion of the Kurdish and Somali migrants being granted a residency permit due to a family reunion, and hence, being offered a health check-up and screening upon arrival. The findings imply a high coverage of infectious disease screening among refugees and asylum seekers in Finland. Acceptability of screening is a prerequisite for achieving a good screening coverage [26]. In this study, a high acceptability of HIV, hepatitis B and C, and syphilis screening was achieved.

Originating from higher prevalence settings, such as Russia, FSU, or Somalia, increases the pre-migratory risk for infection, but the risk also remains elevated after migration. Post-migration infections might be acquired within the destination country or while travelling to a country with a higher disease prevalence or during circulatory migration to the country of origin. Recent evidence suggests an important proportion of post-migration infections arise within the destination countries due to selective sexual mixing and individual risk behaviours [3, 12]. Somali migrants in this study reported relatively low levels of post-migration travelling to the country of origin, which might suggest new infections within the migrant communities after migration. Hepatitis B vaccine is currently included in the childhood vaccination programme in Somalia, but the vaccination coverage has remained modest (42% in 2016) [27]. Unvaccinated migrants from hepatitis B endemic areas should be provided with a hepatitis B vaccine [28]. In Finland, hepatitis B vaccine is recommended free of charge to all individuals, including refugees and asylum seekers, with an increased risk for infection [29]. However, the uptake of the vaccination has not been studied. On the other hand, most Russian origin migrants reported post-migration travelling to the country of origin. Russia is considered an intermediate hepatitis C prevalence country (prevalence between 2.0% and 3.0%), but the prevalence is likely to vary regionally [16].

Usage of health care services was frequent especially among Kurdish and Russian women. Despite frequent consultations, the majority of HBsAg, anti-HCV, and anti-Trpa seropositive cases had been missed. Screening of hepatitis C is not included in the screening guidelines for asylum seekers and refugees in Finland [5], even though evidence shows screening and treatment to be cost-effective [30]. Consequently, the national hepatitis C strategy recommends the screening of migrants from high prevalence countries [31]. On the other hand, the results depict the missing structured screening programme to target non-refugee and non-asylum seeker migrants in Finland. Assuming cost-effectiveness for HBsAg screening at prevalence exceeding 1% [32], our results support the broadening of HBsAg screening to Russian origin migrants in Finland. Health service providers should not miss opportunities to offer information and screening at health care contacts [33].

### Limitations

The Maamu study included Kurdish, Russian, and Somali origin migrants, representing the main migrant groups in Finland [8]. Since health behaviour and risk factors differ significantly between different migrant groups, the results from this study cannot be generalized in allmigrantpopulations. Moreover, despite the population based study design and adoption of population weights in the analyses, additional sources of bias might influence the reliability of the disease burden estimates. A recent study has shown that Russian origin migrants in Finland use cross-border parallel health care services frequently [34]. Only seropositive findings made in Finland are recorded in the NIDR, and hence, the number of missed diagnoses might be an over-estimation, especially among Russian migrants.

Syphilis diagnostics is challenging since a positive syphilis serology (anti-Trpa) can result from either a previously treated infection or latent disease. One-third of the missed diagnoses in the Maamu study had positive syphilis serology (11/33), and six persons with a missed syphilis serology had moved to Finland before 2005. Prior to 2005, the Venereal Disease Research Laboratory (VDRL) test was still commonly used for syphilis screening in Finland [10]. Since VDRL can be negative in latent or previously treated disease, the proportion of missed syphilis serology in this study might be an over-estimate. Nevertheless, every positive syphilis serology finding should be assessed thoroughly.

The quasi-complete separation observed in the multivariate regression model was due to low number of test-positive cases (sparse data). The number of events per variable was 4.25 which can be considered sufficient without causing considerable relative bias or unreliable CI coverage [35].

## Conclusions

In the Maamu study, more than half of chronic hepatitis B and C and syphilis diagnoses had been missed among Kurdish, Russian, and Somali origin migrants in Finland in spite of the national screening recommendations. The prevalence of hepatitis B and C and syphilis was comparable or lower than in previous research among Kurdish, Russian, or Somali origin migrants in high-income countries. No cases of HIV were identified. Our results suggest post-migration spread of hepatitis B among Somali migrants, which could be prevented by enhanced screening and vaccinations. Russian migrants use health services frequently, but are not tested, resulting in missed opportunities to screen for hepatitis C and syphilis. Missed diagnoses expose the migrants to complications, such as cirrhosis, hepatocellular carcinoma, or tertiary syphilis, and may contribute to the continuous spread of infections. The implementation and coverage of current screening strategies among migrants should be evaluated. Targeted hepatitis and syphilis screening alongside prevention strategies should be implemented among migrants upon arrival and in later health care contacts.

## Abbreviations

anti-HCV: Hepatitis C antibodies
anti-Trpa: Treponema pallidum antibodies
CI: Confidence Interval
FSU: Former Soviet Union
HBsAg: Hepatitis B surface antigen
HIV: Human Immunodeficiency Virus
HIVAgAb: HIV antigen and antibodies
Maamu: Migrant population-based health interview and examination survey
NIDR: National Infectious Disease Register
OR: Odds Ratio
PIC: Personal Identity Code
PIS: Population Information System
VDRL: Venereal Disease Research Laboratory test
